# Pulmonary infarction mimicking a lung mass: a case report

**DOI:** 10.11604/pamj.2021.38.127.27595

**Published:** 2021-02-04

**Authors:** Balakrishnan Jayakrishnan, Saif Musabah Al-Mubaihsi, Nisrien Elfatih Elhassan, Rashid Fuad Al-Sukaiti, Jojy George, Younis Said Al-Alawi, Adil Hashim Al-Kindi

**Affiliations:** 1 Department of Medicine, Sultan Qaboos University Hospital, Muscat, Oman,; 2 Department of Radiology, Sultan Qaboos University Hospital, Muscat, Oman,; 3 Department of Nursing, Sultan Qaboos University Hospital, Muscat, Oman,; 4 Division of Cardiothoracic Surgery, Sultan Qaboos University Hospital, Muscat, Oman

**Keywords:** Pulmonary embolism, pulmonary infarction, lung cancer, case report

## Abstract

Pulmonary infarction usually appears as a wedge-shaped opacity with its base placed laterally. Rarely, pulmonary infarctions may appear as a well-defined rounded opacity mimicking lung cancer and surgical lung biopsy may often be required for definitive diagnosis. We report a patient who was admitted with submassive pulmonary embolism who had an incidental finding of a well-defined opacity in computed tomography (CT) scan. The lesion was avid on positron emission tomography (PET) scan and the patient was a smoker. So, we investigated him further with a percutaneous and later a thoracoscopic lung biopsy. Tumour-like pulmonary infarction is often a challenge for the clinicians.

## Introduction

Pulmonary infarction usually appears as a wedge-shaped pleural based opacity with no evidence of air bronchograms [1]. Rarely, pulmonary infarctions may appear as a well-defined rounded opacity mimicking lung cancer [2, 3]. Pulmonary infarctions can also result from a variety of non thromboembolic causes and the radiologic appearance may vary with the underlying cause and temporal evolution of the lesion. Solitary pulmonary nodules can be due to infectious or non-infectious causes and often benign or malignant tumours are considered as the first differentials. We present a case of submassive pulmonary embolism with a focal pulmonary infarct who needed a complete work up as the radiological characteristics mimicked a lung mass.

## Patient and observation

**Figure 1:**
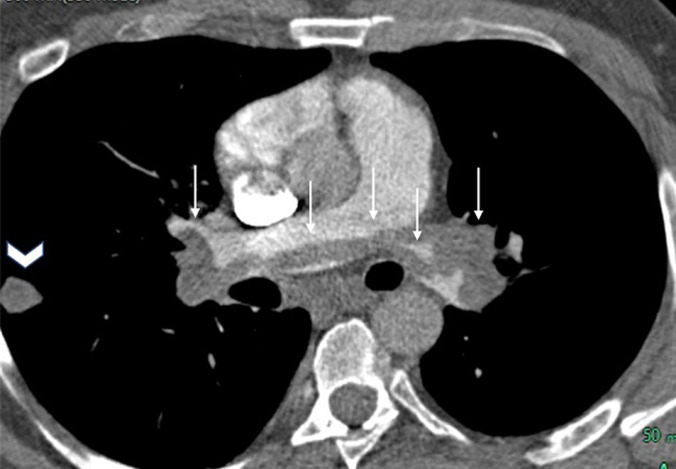
CT pulmonary angiogram mediastinal window axial coronal oblique view demonstrating saddle embolus involving distal main pulmonary artery and extending into both left and right pulmonary arteries ( white arrows) and a well-defined right lung lateral subpleural opacity (white arrow head)

**Figure 2:**
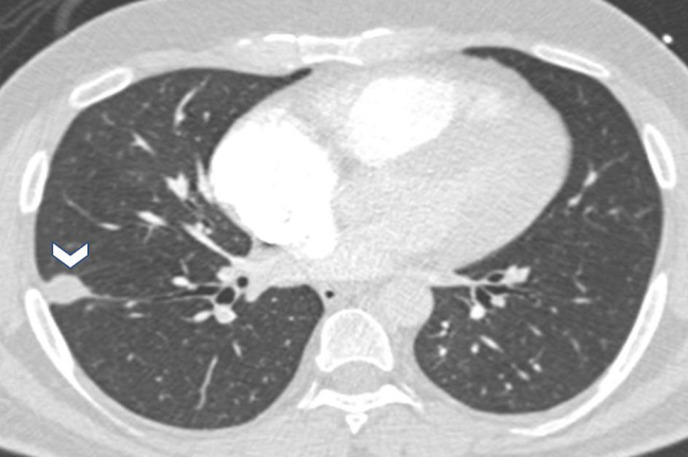
CT pulmonary angiogram axial lung window demonstrating a well-defined right lung lateral opacity (White arrow head)

A 39-year-old male, smoker with generalized anxiety disorder, presented with a two-week history of chest pain and dyspnoea. Hemodynamically he was stable with a pulse rate of 78beats per minute, blood pressure of 130/105mmHg and an oxygen saturation of 87%. Electrocardiogram showed sinus tachycardia and symmetrical T-wave inversions in leads V1 to V4. Cardiac troponin T was elevated, 37ng/L. Chest radiograph was unremarkable. Computed tomography angiogram of pulmonary arteries (CTPA) revealed a large saddle shaped thrombus in main pulmonary artery, extending into the right and left pulmonary arteries as well as to the segmental branches on both sides (Figure 1). In addition, a solitary nodule was seen in the right lower lobe measuring 16 x 10 mm, laterally and just posterior to oblique fissure (Figure 2). Echocardiogram, showed features of right ventricular strain. Pulmonary Embolism Severity Index score was 89 points suggesting Class III, intermediate risk.

**Figure 3:**
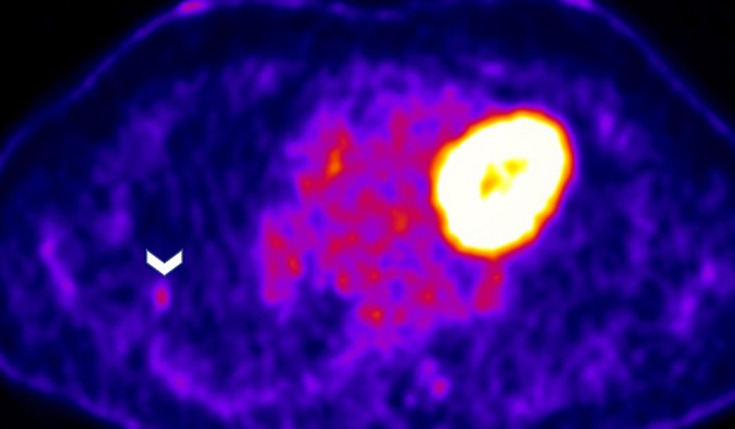
PET CT image that demonstrates a mildly FDG avid right lung lateral subpleural nodule (white arrow head) with SUV of 2.8

Since the patient was not hemodynamically unstable, systemic thrombolysis was not considered. Owing to persistence of symptoms and desaturation, the high thrombus load and the young age, a multidisciplinary team decision was made to proceed with catheter directed thrombolysis. He showed a remarkable response with total disappearance of his symptoms and improvement in oxygen saturation. Anticoagulation was continued with Rivaroxaban. Patient did not have any known predisposing factors for pulmonary embolism (PE). Malignancy being a risk factor, the rounded opacity in the lung of a smoker needed further evaluation. Patient was readmitted after 4 weeks and a CT guided percutaneous biopsy was done. Unfortunately, it was reported as nondiagnostic as it showed only normal tissue without any evidence for granuloma or malignancy. Whole body [18F]-fluorodeoxyglucose (FDG) positron emission tomography-computed tomography (PET/CT) three months later showed the nodule to be FDG avid with a maximum standardized uptake value of 2.8. (Figure 3).

Knowing about a possible mass in his lungs, the patient was very anxious all along. Again, a multidisciplinary decision was made for excision biopsy weighing the surgical risks in this case with the possibility of malignancy. Excision of the nodule was done through video assisted thoracoscopic surgery. Histopathology showed only a localized area of old infarction, 8 mm in maximum dimension without any evidence for malignancy or granuloma. Rivaroxaban was withheld for 48 hours for the percutaneous biopsy and was bridged with unfractionated heparin for the surgical biopsy. Patient was reassured and later had an uneventful follow up in the out-patient clinic.

## Discussion

Here we report a patient who developed a well-defined mass like pulmonary infarct following a submassive PE. The CT characteristics, mildly avid PET finding, smoking status and the chances of PE in malignancy necessitated the need for a lung biopsy. Typical radiographic feature of pulmonary infarction is a wedge-shaped, pleural based opacity without air bronchograms [1]. However, it may also present as a nodule resembling a primary or metastatic lung tumour.

Solitary pulmonary nodules can be due to neoplasms, infectious or non-infectious granulomas, developmental lesions, arterio-venous malformations, hematoma, intrapulmonary lymph node, inflammatory pseudotumour, amyloidoma, rounded atelectasis, mucoid impaction, progressive massive fibrosis, pulmonary artery aneurysm and pulmonary infarct. The diagnosis of tumour like infarction remains a challenge for many reasons [2]. Primarily, pulmonary infarction can closely mimic the radiological features of lung cancer [3]. Pulmonary thrombus seen as a solitary pulmonary nodule on a chest radiograph has also been reported [4]. Even the PET scan can show an increased uptake [5]. Moreover, cytological changes in pulmonary infarctions may also produce malignant-appearing cells in respiratory secretions and aspiration biopsy specimens [6]. Our patient had a well-defined rounded, fairly large opacity which was also FDG avid.

Occlusion of a pulmonary artery usually does not produce tissue necrosis as the lungs receive oxygen from bronchial arteries and the airways as well. The incidence of pulmonary infarction in patients with pulmonary embolism is usually 10%, but may go up to 30% [7]. Predisposing factors include left heart failure, accompanying pneumonia, sepsis, malignancy, advanced age and a higher clot burden. Infarction is more common with emboli that are distal rather than proximal [8]. Non-thromboembolic causes of pulmonary infarctions include pulmonary infections, diffuse alveolar damage, pulmonary torsion, lung cancer, amyloidosis, bronchial artery embolization therapy, vasculitis, Swan-Ganz catheter use, or sickle-cell disease [9].

Finally, round pneumonia should also be entertained in the differential diagnosis of pulmonary infarct and lung tumour [10]. Nevertheless, persistence of the lesion without any change in size for 5 months, from presentation to the time of lung biopsy, was not in favour of an acute infective process. On the other hand, some pulmonary infarctions are known to change only slowly over time.

## Conclusion

Our patient with a submassive pulmonary embolism developed a pulmonary infarct, the characteristics of which resembled a lung mass. Since he was a smoker and the lesion was PET avid, a lung biopsy was done. In spite of advancements in diagnostic methods tumour like pulmonary infarction will continue to pose a challenge for the clinicians. Often, a definitive surgical intervention is needed for a proper diagnosis.
